# Beyond a Single Clock: Integrating Injury-to-Flap and Fixation-to-Flap Timing in Severe Open Lower-Limb Trauma

**DOI:** 10.7759/cureus.117902

**Published:** 2026-10-14

**Authors:** Daniel Gravino, Kyle Debono

**Affiliations:** 1 Plastic and Reconstructive Surgery, Mater Dei Hospital, Msida, MLT; 2 Surgery, Mater Dei Hospital, Msida, MLT

**Keywords:** fixation-to-flap, fracture-related infection, free flap, lower-limb reconstruction, microsurgery, negative-pressure wound therapy, open tibial fracture, orthoplastic surgery, soft-tissue coverage

## Abstract

Severe open lower-limb fractures requiring flap reconstruction demand coordinated skeletal stabilization and durable soft-tissue coverage. Injury-to-flap timing remains central to care, and current British guidance recommends definitive coverage within 72 hours, with definitive internal fixation accompanied by definitive coverage. Observational evidence increasingly examines a second interval, from definitive fixation to flap reconstruction, but its incremental value and optimal threshold remain uncertain.

This narrative review integrates historical studies, contemporary comparative cohorts, systematic reviews, and guidelines, supplemented by targeted literature verification during revision. Several studies associate shorter injury-to-flap or fixation-to-flap intervals with fewer infections. Other cohorts report nonsignificant timing differences or favorable outcomes after selected staged procedures. These findings address different populations, timing origins, and outcomes; they neither establish equivalent safety of delay nor justify relaxing established standards. Confounding by injury severity, physiological instability, wound preparation, and access to specialist care remains substantial. Negative-pressure wound therapy and local antimicrobial strategies are adjuncts, with no demonstrated capacity to neutralize the consequences of delayed coverage.

Both intervals should be recorded within a coordinated orthoplastic pathway. Early coverage remains the goal, while clinically necessary deviations require explicit documentation and prompt reassessment. Fixation-to-flap time may identify additional opportunities for improving coordination, but it is not a validated replacement deadline or an unadjusted institutional performance measure. Prospective studies should capture treatment intent, reasons for delay, granular injury severity, consensus-defined fracture-related infection, and long-term functional outcomes.

## Introduction and background

Severe open lower-limb fractures combine skeletal instability with contamination, tissue devitalization, and exposure of bone or fixation material. Gustilo-Anderson type IIIB describes extensive soft-tissue loss with periosteal stripping and exposed bone requiring reconstructive coverage; type IIIC denotes an associated arterial injury requiring repair, regardless of the soft-tissue grade. Flap reconstruction transfers living tissue with its blood supply to cover a defect: a pedicled flap retains its vascular connection, whereas a free flap requires microsurgical vascular anastomoses. These injuries require integrated orthopedic and plastic surgical care [1].

Godina's 532 extremity reconstructions established the association between reconstruction within 72 hours and favorable outcomes [2]. Gopal et al. developed the coordinated "fix and flap" approach, combining wound excision, skeletal stabilization, and vascularized coverage [3]. These historical observations inform, but are distinct from, current clinical standards. The British Orthopedic Association Standards for Trauma (BOAST) recommend definitive closure or coverage within 72 hours of injury when not achieved during debridement, and definitive internal stabilization only when immediately followed by definitive soft-tissue cover [4]. National Institute for Health and Care Excellence (NICE) guideline NG37 similarly recommends combined orthoplastic care and concurrent internal fixation and definitive coverage [5].

Contemporary reviews reach different statistical conclusions about timing [6-9]. This uncertainty concerns the magnitude, independence, and generalizability of timing effects; it does not suspend guideline recommendations. Injury-to-flap and definitive-fixation-to-flap intervals also measure different parts of care. Fixation and coverage together on day five differ from fixation on day one and coverage on day five, although both patients exceed the 72-hour benchmark. This narrative review asks whether fixation-to-flap timing adds clinically useful information beyond injury-to-flap timing, and how patient condition, wound readiness, treatment strategy, and system capability should shape its interpretation.

## Review

Literature selection

The references cited in the initial manuscript and reviewer-nominated sources formed the starting literature set. Targeted web-assisted searches and source verification were undertaken during revision, with the final verification performed on October 9, 2026. Sources comprised PubMed/MEDLINE-indexed records retrieved through web searches, PubMed Central, publisher websites, and official BOAST and NICE guidance. Search concepts combined open fracture or tibial fracture with flap, timing, definitive fixation, fix and flap, orthoplastic, and infection; exact-title and author searches were used to locate and verify individual reports. Adjunct searches addressed negative-pressure wound therapy (NPWT), local antibiotics, dead-space management, fracture-related infection (FRI), and functional outcomes. Reference lists and included-study tables in accessible reports were checked to identify additional sources and overlapping evidence.

Selection was purposive and relevance-based, including English-language human clinical studies and evidence syntheses available by the search date that addressed acute traumatic lower-limb reconstruction, timing, or important pathway modifiers. Historical studies were retained without a lower publication-date limit. No minimum sample size was imposed; small uncontrolled series were used only to illustrate feasibility. Pediatric-only, upper-limb-only, nontraumatic reconstruction, animal studies, isolated case reports, and duplicate conference reports were not used as core timing evidence. Broader fracture populations and adjunct trials were retained when informative, with their indirectness identified.

Selection and extraction were undertaken as a single narrative synthesis rather than through independent duplicate screening. This was a targeted literature update, not an exhaustive systematic database search; Embase, Scopus, and the Cochrane Library were not independently searched. Accessible full texts were preferred, and abstract-based extraction is identified in the tables. Studies were compared qualitatively by design, population, timing, origin, outcome definition, follow-up, adjustment, and precision. No formal study-level risk-of-bias instrument, pooled analysis, or formal overlap coefficient was applied. Reporting was informed by the Scale for the Assessment of Narrative Review Articles (SANRA), without assigning a formal score [10].

Scope and operational definitions

The principal population is severe open lower-limb trauma requiring flap coverage, especially IIIB and IIIC tibial fractures. Mixed lower-extremity cohorts, free-flap-only series, and cohorts including direct closure or grafting are identified separately. Definitive coverage means durable closure protecting the underlying injury; when exposed structures require vascularized tissue, an interim dressing is not definitive coverage.

Injury-to-flap time runs from the injury to definitive flap reconstruction. Fixation-to-flap time runs from skeletal fixation intended to provide stability through fracture healing to definitive flap reconstruction. Temporary stabilization does not start the latter interval. Intent, rather than implant type, determines the classification: an external fixator may be temporary or definitive. When a construct initially intended as definitive is revised, both procedures and the original treatment intent should be recorded rather than resetting the interval without explanation. Same-operation fixation and coverage have no intervening operative stage, although calendar-day records may conceal several hours of separation between distinct procedures.

Clinically necessary staging should be distinguished from a scheduled pathway that still contains remediable delay. Physiological instability, further necessary wound excision, or vascular treatment may prevent immediate reconstruction. Operating-room shortages, delayed referral, or lack of team availability are system constraints that warrant escalation. Calling a pathway "planned" does not establish that its delay was unavoidable or safe.

Reassessing injury to flap timing

Godina's historical findings support early reconstruction but arose in a different treatment era and cannot isolate timing from case selection and accompanying care [2]. Later studies examined longer intervals. D'Alleyrand et al. evaluated 69 patients requiring 74 flaps and found increasing adjusted odds of complications after day seven, without identifying an increase during days one to seven [11]. Lee et al. studied 358 free flaps and reported fewer major complications within three days than at four to 90 days; the absence of detected differences between three days or less and four to nine days did not constitute an equivalence test [12]. These studies explore possible risk gradients; they do not validate routine postponement to seven or ten days.

Tiongco et al. included 16 studies and 973 patients. Coverage within 72 hours was associated with lower infection risk (risk ratio (RR) 0.48, 95% confidence interval (CI) 0.25-0.89) and fewer overall complications (RR 0.59, 95% CI 0.36-0.99) [6]. These are relative reductions of 52% and 41%, not percentage-point differences. Comparative RR pooling differs from the proportional meta-analysis and meta-regression used by Alves et al. across 26 studies and 451 free flaps [7]. Alves et al. detected no significant difference in infection or flap failure between four timing categories. Comparing proportions across different studies and case mixes cannot establish equivalence of early and delayed treatment.

Dehnadi et al. pooled 14 studies and 2,652 patients: infection RR 0.69 (95% CI 0.47-1.02; p = 0.064) and osteomyelitis RR 0.28 (95% CI 0.16-0.49) [8]. Included fixation-based cohorts complicate interpretation as a purely injury-based comparison. Ruprecht et al. reported an infection odds ratio (OR) of 0.34 (95% CI 0.23-0.52) across five studies and 454 IIIB/IIIC tibial injuries, using early-treatment cutoffs of 72-168 hours [9]. Table 1 compares the review methods and their limitations.

**Table 1:** Contemporary systematic reviews of reconstructive timing All numerical intervals in the effect column are 95% CIs. Reviews share some primary reports; totals must not be summed. Published infection labels are retained and are not assumed to meet consensus FRI criteria. No new pooled analysis was performed. CI - confidence interval; FRI - fracture-related infection; OR - odds ratio; RR - risk ratio.

Review	Design and population	Clock and synthesis	Outcome and follow-up	Reported effects with 95% CI	Main limitation
Tiongco et al. [6]	16 studies; 973 patients; mixed lower-limb open fractures; predominantly free flaps.	Injury to coverage; 72-hour and 7-day comparisons; random-effects comparative RR pooling.	Study-defined infection and complications; 10 studies reported at least 12 months of follow-up.	Within 72 h: infection RR 0.48 (0.25-0.89); overall complications RR 0.59 (0.36-0.99).	Observational case mix; variable definitions; some non-flap reconstruction and pediatric cases.
Alves et al. [7]	26 studies; 451 free flaps; Gustilo III lower-limb fractures.	Injury to flap; <72 h, 72 h-1 week, 1-6 weeks, >6 weeks; proportional pooling and meta-regression.	Infection and flap failure; follow-up varied.	Category tests: infection p = 0.3331; flap failure p = 0.0772. No common adjusted timing OR or RR.	Cross-study proportions; small categories and wide pooled CIs; no equivalence test.
Dehnadi et al. [8]	14 studies; 2,652 patients; mixed lower-limb open fractures.	Framed as 72 h or earlier versus later; random-effects comparative RR pooling; clock origins vary.	Study-defined infection and osteomyelitis; follow-up varied.	Infection RR 0.69 (0.47-1.02), p = 0.064; osteomyelitis RR 0.28 (0.16-0.49).	Serious or critical bias in included cohorts; infection heterogeneity I² = 64.5%; mixed clocks.
Ruprecht et al. [9]	5 studies; 454 patients; IIIB/IIIC tibial fractures.	Early versus late coverage; thresholds 72-168 h; comparative OR pooling.	FRI as labeled by contributing studies; harmonized definition and follow-up not verified.	Infection OR 0.34 (0.23-0.52), p < 0.001.	Small evidence set; variable cutoffs; extraction limited to accessible abstract.

Confirmed overlap includes Kang (2020), Lee JH (2012), Rezzadeh (2015), and Rinker (2008) in both Tiongco et al. and Alves et al., and Kloeters (2017), Liu (2012), and Lee SY (2021) in both Tiongco et al. and Dehnadi et al. [6-8]. Shared reports reduce the independence of review-level findings, although the cohorts or subgroups extracted may differ. The complete eligible-study set for Ruprecht could not be verified from the accessible report, so an exhaustive four-review overlap matrix is not claimed.

Coordination and fixation to flap timing

Mathews et al. reported deep infection in 2/48 fractures treated with fixation and coverage in one operation versus 9/26 treated separately, or 4.2% versus 34.6% [13]. The crude absolute difference is 30.4 percentage points; it is not an adjusted treatment effect. Selection for one-stage care may reflect less complex injury or better physiological readiness. Aljawadi et al. described favorable results in 102 patients managed with combined reconstruction at a mean of 7.71 days after injury [14]. An uncontrolled successful pathway cannot establish that delaying an otherwise ready patient produces the same outcome.

Kuripla et al. analyzed 296 Gustilo III tibial fractures at 14 trauma centers. Fixation-to-flap time had the strongest association with infection among the timing variables in their multivariable model (OR 1.04 per additional day, 95% CI 1.01-1.08; regression n = 260) [15]. Under a constant log-linear model, 1.04 raised to the seventh power corresponds to approximately 32% higher odds over seven days; this illustrates the model, not an observed absolute risk increase. The Supporting Plastics and Orthopedics Alliance in Reducing Trauma Adverse Events (SPARTA) cohort included 373 fractures. The reported RR for coverage up to two days after fixation was 1.12 (95% CI 0.92-1.37; p = 0.26); RRs beyond two and five days were 1.59 and 1.64, respectively, without CIs in the accessible abstract [16]. Adjustment included time to debridement and early antibiotics. Data-selected thresholds and residual confounding prevent these findings from defining a universally safe staging window.

In Le et al.'s cohort of 863 fractures, surgical-site infection (SSI) was reported in 16.6%, 16.7%, and 28.8% across progressively later fixation-to-coverage groups [17]. The adjusted estimates in the abstract concern all-cause complications, not specifically infection, and must be labeled accordingly. Parlamas et al. reported nonsignificant categorical infection differences across three intervals after fixation (p = 0.40), but a significant timing term in multivariable analysis (p = 0.04) [18]. Khan et al.'s 52-extremity series also showed an imprecise, nonsignificant difference across a 72-hour fixation-based threshold [19]. Neither finding identifies a reliable breakpoint.

Conover et al. assessed 1066 IIIB tibial fractures using insurance claims and found increasing one-year complication incidence with longer fixation-to-flap intervals [20]. Koltenyuk et al. examined 1970 open lower-extremity fractures and reported an adjusted OR of 1.091 per additional day (95% CI 1.047-1.138) for inpatient FRI [21]. This represents 9.1% higher odds, not a 9.1-percentage-point increase in infection probability. It is conditional on the fitted model, does not prove a causal daily penalty, and should not be extrapolated across unobserved durations. Claims coding, incomplete clinical detail, and, for Koltenyuk et al., index-admission-only ascertainment constrain interpretation. Table 2 distinguishes adjusted estimates from crude comparisons.

**Table 2:** Selected clinical studies of timing and sequencing NR* indicates that the detail or CI was not reported in the accessible abstract or report used for extraction, rather than necessarily absent from the full publication. Missing values were not imputed. Effect estimates are identified as adjusted, pooled, or crude; descriptive rate differences are unadjusted. Le et al. describe the intermediate interval as three to seven days in 'Methods' and four to seven days in 'Results'; the latest interval is labeled 7+ days. CI - confidence interval; FRI - fracture-related infection; IMN - intramedullary nail; NPWT - negative-pressure wound therapy; OR - odds ratio; ORIF - open reduction and internal fixation; RR - risk ratio; SPARTA - Supporting Plastics and Orthopedics Alliance in Reducing Trauma Adverse Events; SSI - surgical-site infection

Study and design	Population and coverage	Clock and comparison	Infection endpoint	Follow-up	Reported estimate and 95% CI	Main limitation
D'Alleyrand 2014 [11] Retrospective single-center	69 patients; 74 flaps for tibial shaft, plateau, or pilon injuries.	Injury to flap; modeled days 1-7 separately from later days.	Infection or other flap complication requiring surgery; detailed infection criteria NR*.	NR*	Adjusted odds increased 11% for complications and 16% for infection per day after day 7; p < 0.04. CIs NR*.	Small cohort; nonlinear threshold derived within the dataset. Abstract-limited extraction.
Lee et al. 2019 [12] Retrospective single-center	358 free flaps for below-knee trauma, 1979-2016.	Injury to flap: ≤3, 4-90, >90 days; delayed group subdivided at day 10.	Flap complications and failure; separate infection criteria NR*.	NR*	Major complications ≤3 vs 4-90 days: OR 0.40; p = 0.04. For 4-9 vs 10-90 days, flap failure RR 0.29; p = 0.025. CIs NR*; adjustment not verified.	Long treatment era; data-derived cutoff; no equivalence design. Abstract-limited extraction.
Mathews et al. 2015 [13] Retrospective single-center	73 patients; 74 grade III open tibial fractures; flap/coverage distribution NR*.	Same-operation fixation and cover (48) versus separate operations (26); also injury-based 72 h.	Deep infection; detailed criteria NR*.	At least 1 year	Crude deep infection 2/48 (4.2%) vs 9/26 (34.6%); difference 30.4 percentage points. Adjusted effect and CI NR*.	Strong selection-by-readiness risk; coverage population not fully characterized in abstract.
Kuripla et al. 2021 [15] Retrospective multicenter	296 Gustilo III tibial fractures requiring flaps at 14 centers; regression n = 260.	Injury, debridement, and definitive fixation to flap compared in models.	Wound infection; detailed adjudication criteria NR*.	NR*	Adjusted fixation-to-flap OR 1.04 per day (1.01-1.08); p = 0.02. Overall infection 96/296 (32.4%).	Residual confounding; smaller regression sample; abstract-limited endpoint detail.
SPARTA 2023 [16] Retrospective international cohort	373 open tibial shaft fractures requiring flap coverage.	Definitive fixation to flap; day 0 and 2- and 5-day cutoffs.	Deep infection; detailed criteria NR*.	NR*	Reported RR for interval up to 2 days 1.12 (0.92-1.37); p = 0.26. Beyond 2 days RR 1.59; beyond 5 days RR 1.64; CIs NR* for latter estimates.	Cutoffs selected from observed curves; adjustment included debridement and early antibiotics. Not a validated safe window.
Le et al. 2024 [17] Retrospective claims cohort	863 open lower-extremity fractures; free or axial flaps after ORIF.	Fixation to flap; 0-3 days, intermediate interval, and 7+ days as labeled in the abstract.	Claims-coded SSI and acute osteomyelitis.	90 days	Crude SSI: 16.6%, 16.7%, 28.8%. Reported adjusted all-complication ORs: 0.22 (0.15-0.32) and 0.26 (0.17-0.40), earlier groups versus latest.	Adjusted ORs concern all complications. The abstract labels the intermediate bin inconsistently; exact boundaries were not inferred.
Parlamas et al. 2026 [18] Retrospective single-center	274 Gustilo III lower-extremity fractures; flap composition included in model.	Fixation to flap: within 24 h, 24-72 h, >72 h; continuous timing also modeled.	Wound infection; detailed criteria NR*.	NR*	Crude rates 23.2%, 25.0%, 31.5%; p = 0.40. Timing term p = 0.04 in multivariable model; adjusted effect size and CI NR*.	Several correlated timing variables; coefficients required to judge magnitude and precision. Abstract-limited extraction.
Khan et al. 2025 [19] Retrospective single-center	51 patients; 52 IIIB/IIIC tibial extremities; 30 free and 22 local flaps.	Definitive fixation to flap: 72 h or earlier versus later.	Deep SSI; detailed diagnostic criteria NR*.	NR*	Crude infection 4/22 (18%) vs 9/30 (30%); p = 0.33. Adjusted timing estimate and CI NR*.	Small subgroups; flap choice linked to injury complexity; no equivalence inference.
Conover et al. 2026 [20] Retrospective claims cohort	1066 IIIB tibial fractures; flap coverage within 45 days of fixation.	Injury to fixation and fixation to flap; 3-day groups and daily regression.	Claims-coded SSI; also wound disruption and reoperation.	1 year	Reported SSI incidence slope +0.53 percentage points/day; p < 0.001. CI and patient-level adjusted timing OR NR*.	Incidence regression is not an individual causal risk increment; limited wound-level data.
Koltenyuk et al. 2026 [21] National Inpatient Sample	1970 open tibial, fibular, or femoral fractures requiring flaps; graft-only cases excluded.	Admission and definitive fixation to flap; days modeled continuously.	Inpatient coded FRI; not verified consensus adjudication.	Index admission only	Adjusted fixation-to-flap OR/day 1.091 (1.047-1.138); p < 0.001. Overall inpatient FRI 9.9%.	Misses postdischarge infection; temporal sequence and residual confounding constrain causal interpretation.
Sreedharan et al. 2024 [22] Observational single-center	100 salvaged tibial fractures; 74 flaps and 26 grafts/direct closures.	Median injury-to-reconstruction 5.5 days; fixation-to-reconstruction 3 days.	Clinical wound features and positive culture.	Mean 20.8 months	Unadjusted timing comparisons nonsignificant; injury-to-coverage versus infection p = 0.48. No adjusted timing estimate.	Mixed reconstruction population; near-universal NPWT cannot test its interaction with delay.
Morris et al. 2025 [23] Retrospective single-center	149 IIIB/IIIC tibial fractures; 34 immediate IMN and 115 initial external fixation.	Immediate IMN then delayed flap versus temporary external fixation then combined IMN and flap.	Bone infection confirmed by positive tissue cultures.	At least 6 months	Adjusted deep-infection OR 1.14 (0.46-2.85); p = 0.780.	Wide CI; compares fixation strategies, not randomized timing. No proof of equivalent safety.
Nishida et al. [24] Retrospective case series	10 severe open-fracture cases treated by an orthoplastic team.	Definitive internal fixation followed by staged free-flap coverage.	Deep infection.	NR*	Complete flap survival and union; no deep infection. No comparative effect estimate.	No control group; ten selected cases cannot establish equivalence or a safe timing threshold.

What selected staged cohorts can establish

Sreedharan et al. reported 100 primarily salvaged open tibial fractures, comprising 56 free flaps, 18 local or pedicled flaps, 14 skin grafts, and 12 direct closures [22]. Median injury-to-reconstruction and fixation-to-reconstruction intervals were 5.5 and three days. Their unadjusted comparisons did not detect associations with infection or union; they did not establish an independent null timing effect. The mixed reconstruction population also limits applicability to flap-requiring IIIB/IIIC injuries.

Morris et al. compared immediate intramedullary nailing followed by delayed coverage with initial external fixation followed by combined nailing and coverage in 149 IIIB/IIIC tibial fractures. The adjusted OR for deep infection was 1.14 (95% CI 0.46-2.85; p = 0.780) [23]. This CI remains compatible with substantial benefit or harm and does not establish noninferiority. Nishida et al. reported complete flap survival and union without deep infection in ten staged cases, without a contemporaneous comparator [24]. Neither that series nor the 100-patient Sreedharan cohort provides a prespecified equivalence margin and sufficiently precise comparative estimate to establish equal safety. Successful salvage in selected patients therefore supports feasibility when ideal timing is unattainable, rather than elective separation of internal fixation and coverage contrary to current guidance.

Wound preparation and contemporary adjuncts

Adequate excision of nonviable and contaminated tissue remains a prerequisite to reconstruction [1,4,5]. A timing target should not encourage coverage of retained devitalized tissue, but serial excision should have a documented clinical indication rather than become a routine explanation for delay. Operative assessment should describe residual contamination, tissue viability, dead space, vascular status, and the next definitive step. Readiness is a clinical judgment that should be recorded and reassessed; it is not yet a validated quantitative variable.

The Wound Management of Open Lower Limb Fractures (WOLLF) randomized trial enrolled 460 patients and did not demonstrate better 12-month disability or significantly fewer deep SSIs with NPWT than with standard dressings. Deep SSI occurred in 7.1% versus 8.1%; the reported difference favoring NPWT was 1.0 percentage point (95% CI -4.2 to 6.3; p = 0.64) [25]. This trial evaluated dressings, not a policy of extending time to coverage.

Barone et al.'s 34-study review describes NPWT as a temporizing strategy in 804 patients [26]. Rocks et al. reported associations with lower flap-failure rates, without a significant deep-infection reduction [27]. These observational syntheses cannot show that NPWT neutralizes delay. In particular, the 98% NPWT exposure in Sreedharan et al. leaves no meaningful comparison for testing NPWT-by-timing effect modification [22]. NPWT may manage an open wound while reconstruction is arranged; it should not become a reason to postpone coverage.

Antimicrobial prophylaxis and dead-space management also influence infection risk. BOAST recommends intravenous prophylaxis as soon as possible, ideally within one hour of injury, using an agreed network policy [4]. Antibiotic selection, redosing, contamination-specific requirements, and administration times should therefore be captured in timing studies. Local delivery is an adjunct to systemic prophylaxis and surgical source control.

The randomized intrawound vancomycin (VANCO) trial analyzed 980 patients with high-risk tibial plateau or pilon fractures and reported deep infection probabilities of 6.4% with intrawound vancomycin and 9.8% without it at 182 days (risk difference -3.4 percentage points, 95% CI -6.9 to 0.1; p = 0.06). A post hoc gram-positive analysis favored vancomycin, but IIIB and IIIC injuries were excluded [28]. Its findings cannot be directly transferred to the present flap-requiring population. Patterson et al.'s 113-patient IIIB tibial cohort associated an antibiotic bead pouch with fewer FRIs requiring debridement or amputation than NPWT-based strategies [29]. This nonrandomized comparison supports further investigation of local antimicrobials and management of dead space, not a longer permissible coverage interval.

Injury severity and system capability

Gustilo-Anderson grading should be supplemented by more granular injury description. The Orthopedic Trauma Association (OTA) Open Fracture Classification separately records skin, muscle, arterial injury, contamination, and bone loss [30]. In 2215 fractures, Kovvur et al. demonstrated that these domains do not align uniformly with Gustilo-Anderson grades, particularly for contamination [31]. Subsequent multicenter work links individual domains to treatment requirements [32]. These characteristics can influence both the need for staging and the risk of adverse outcomes, leaving residual confounding when only a broad fracture grade is adjusted for.

Resources also shape treatment. Tiondi et al. documented limitations in fracture-fixation resources in low- and middle-income settings [33], while Otieno et al. demonstrated selected free-flap salvage at equipped East African tertiary centers [34]. These findings concern feasibility and access, not an alternative biological deadline. Resource constraints should prompt a documented interim plan, early referral, and escalation of barriers. A scheduled delay caused by unavailable operating capacity remains potentially modifiable even when temporary wound care is well organized.

A contemporary orthoplastic decision framework

The clinical pathway begins with resuscitation, limb assessment, prompt systemic antibiotic prophylaxis, and urgent treatment of vascular compromise. Physiological instability and immediate limb-threatening problems determine the first intervention. Consultant orthopedic and plastic surgeons should plan wound excision, skeletal stabilization, and coverage together; the injury-based 72-hour target remains the benchmark throughout this process [4,5].

Once physiology and wound preparation permit definitive reconstruction, fixation and coverage should be coordinated in the same operation. Definitive internal fixation should only proceed when definitive cover can accompany it. When further clinically necessary excision, physiological recovery, or transfer prevents this, temporary stabilization and a defined interim wound-management plan preserve the route to definitive treatment. An external construct intended to remain through healing should be recorded as definitive, irrespective of its appearance [4,5].

If definitive fixation has already been performed without coverage, the fixation-to-flap interval should trigger prompt specialist reassessment and coordination of the earliest feasible cover. It does not reset the injury clock. When the target cannot be achieved, the record should state the clinical or organizational reason, responsible team, next reassessment, interim wound treatment, and anticipated definitive operation. Delayed referral or unavailable theater capacity requires escalation even when the operation has been scheduled.

The framework prioritizes physiology, wound readiness, fixation intent, coordinated coverage, interim wound care, and system capability. It is a clinical interpretation of the evidence and existing guidance, not a validated prediction rule or an alternative timing recommendation. Neither fixation nor coverage should be postponed to make a process interval appear compliant.

Interpreting the evidence and measuring quality

The principal limitations are confounding by indication, inconsistent timing origins, heterogeneous populations and outcomes, and incomplete reporting. Earlier reconstruction may mark a better-resourced pathway; greater injury severity may cause both delay and infection. Infection arising before reconstruction may itself postpone coverage, creating reverse causation. Analyses confined to patients eventually receiving a flap may also omit early amputations or deaths. These selection processes are not resolved by statistical significance or a large administrative sample.

Injury-to-flap, admission-to-flap, debridement-to-flap, and fixation-to-flap intervals are not interchangeable. Similarly, superficial SSI, deep infection, osteomyelitis, and consensus-defined FRI cannot be treated as identical endpoints. The Metsemakers et al. consensus distinguishes confirmatory from suggestive features, providing a more reproducible foundation for prospective FRI ascertainment [35]. Neither legacy clinical labels nor diagnosis codes should retrospectively be presented as equivalent to adjudicated consensus-defined FRI without evidence.

Nonsignificant comparisons do not establish equivalence, and adjusted associations remain dependent on measured covariates and model specification. Broad time categories can conceal nonlinear relationships; data-selected cutoffs may appear more certain than they are. Comparative effect-size pooling and proportional meta-analysis address different questions. Several primary cohorts recur in the reviews, so the reviews and their participant totals must not be treated as independent evidence. A new pooled analysis or complete four-review overlap calculation was not undertaken.

Fixation-to-flap time is therefore a useful candidate process measure alongside the guideline-based injury clock, rather than a substitute performance target. Audit should show both intervals, adherence to established standards, treatment intent, documented reasons for deviation, and outcomes. Risk adjustment is essential before comparing centers. A proposed “time from readiness to coverage” measure could identify remediable delay, but would require reproducible readiness definitions and prospective validation. It should not be used to retrospectively excuse missed targets.

The review itself is limited by purposive selection, English-language restriction, incomplete full-text access, and the absence of duplicate screening or formal risk-of-bias assessment. Its search should not be interpreted as demonstrating completeness. Study-specific access limitations are identified in Tables 1 and 2; absent details and confidence intervals were not imputed. These constraints strengthen the case for cautious interpretation rather than a new universal deadline.

Future directions

Prospective multicenter registries should record injury, antibiotic administration, first and final wound excision, each temporary or definitive fixation procedure, and definitive coverage using actual timestamps. They should distinguish reasons for delay, document changes in fixation intent, and capture injury domains, vascular treatment, local antibiotics, NPWT exposure, and center-level resources. Analyses should prespecify timing origins and covariates, consider nonlinear effects and time-varying confounding, and establish whether infection preceded or followed the relevant interval.

Follow-up should extend beyond the index admission and include consensus-defined FRI, union, reoperation, secondary amputation, mobility, pain, return to work, and validated patient-reported outcomes. The Lower Extremity Assessment Project (LEAP) demonstrated the importance of sustained functional assessment after limb-threatening trauma [36]. It informs outcome selection, not a causal comparison of reconstructive timing. Studies should include different health systems and compare care pathways with adequate adjustment, while maintaining early coordinated reconstruction as the clinical goal.

## Conclusions

Injury-to-flap and fixation-to-flap timing describe complementary dimensions of severe open lower-limb fracture care. Current evidence supports early, coordinated reconstruction and does not justify replacing the established 72-hour benchmark with a new fixation-based deadline. Favorable outcomes after selected staged care demonstrate feasibility, not equivalent safety of delay. Neither NPWT nor local antibiotics have been shown to offset postponement of definitive coverage. Both intervals should be recorded, clinically necessary deviations documented, and system barriers addressed. The next step is prospective evaluation of complete orthoplastic pathways with granular injury adjustment and long-term functional outcomes.
